# Molecular Dynamics Simulation of the Influence of Nanoscale Structure on Water Wetting and Condensation

**DOI:** 10.3390/mi10090587

**Published:** 2019-08-31

**Authors:** Masaki Hiratsuka, Motoki Emoto, Akihisa Konno, Shinichiro Ito

**Affiliations:** Department of Mechanical Engineering, Kogakuin University, Tokyo 163-8677, Japan

**Keywords:** functional surface, condensation, molecular dynamics, wettability, nanoscale structure

## Abstract

Recent advances in the microfabrication technology have made it possible to control surface properties at micro- and nanoscale levels. Functional surfaces drastically change wettability and condensation processes that are essential for controlling of heat transfer. However, the direct observation of condensation on micro- and nanostructure surfaces is difficult, and further understanding of the effects of the microstructure on the phase change is required. In this research, the contact angle of droplets with a wall surface and the initial condensation process were analyzed using a molecular dynamics simulation to investigate the impact of nanoscale structures and their adhesion force on condensation. The results demonstrated the dependence of the contact angle of the droplets and condensation dynamics on the wall structure and attractive force of the wall surface. Condensed water droplets were adsorbed into the nanostructures and formed a water film in case of a hydrophilic surface.

## 1. Introduction

With recent advances in the micro- and nanoscale processing and measurement technology, it has become possible to add fine structures to surfaces [1,2,3]. These microstructures are known to have significant effects on wettability of liquids [4,5,6] and are expected to be able to control water–surface interactions and wettability by changing the size of wall structures [7,8]. Wettability of metals and nanostructures changes the friction of objects, chemical reaction on the surface, and crystallization of proteins [9,10,11,12,13,14,15]. Mirco-nanosurface is also expected to be used as a highly efficient heat transport device. In case of the condensation heat transfer, micro-nanostructure of a condensation surface is quite essential for achieving a high heat transfer [16]. The condensation growth morphology depends on micro-nanoscale surface topography [17,18]. Also, the condensation form, filmwise and dropwise condensation, is controlled by the surface structures [19,20]. For this reason, the impact of surface structure and wettability on the condensation characteristics has been experimentally investigated [21,22,23,24,25,26,27,28,29]. However, it is still difficult to observe the initial stage of liquid condensation on nanoscale surfaces directly and analyze the mechanism of the observed phenomena using experimental methods alone. Wettability is affected not only by the shape and size of asperities but also the molecular scale crystal structure of materials [30,31]. Therefore, detailed observations at the atomic scale are required to understand the mechanism of condensation on nanoscale structures.

**Figure 1:**
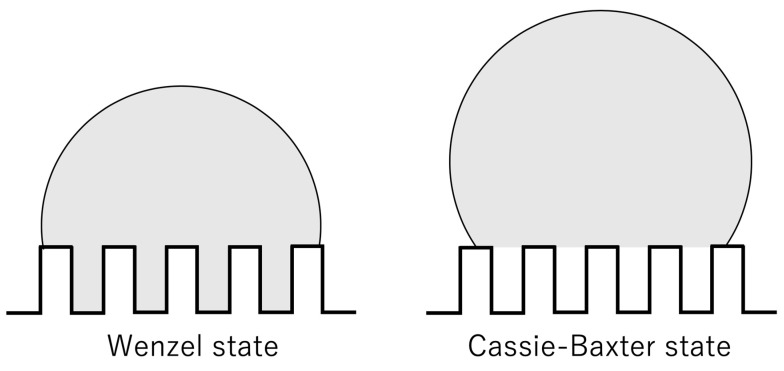
Schematic diagrams of Wenzel state and Cassie-Baxter state.

Analysis using a molecular simulation is one way to elucidate such nanoscale phenomena [32,33]. In previous studies, wettability and the contact angle of droplets with nanoscale surfaces were analyzed using molecular dynamics simulations [6,34,35]. They demonstrated that the Wenzel state [36] and Cassie-Baxter state [37] can be observed depending on the size and spacing of nanostructures, as well as parameters of the molecular interactions between water and surface molecules. The schematic diagrams of the Wenzel state and Cassie-Baxter state are shown in Figure 1. Larger adsorption energy between a wall and water puts the former in the Wenzel state. Also, the smaller the height of a structure, the lower the gap between its wall and droplets, which puts the structure in the Wenzel state [34]. While molecular dynamics calculations have been performed for droplets on nanosurfaces, there are not enough studies focusing on condensation, except only a few investigating condensations on nanostructures under limited conditions [38,39]. These latter studies analyzed the temperature change during condensation and heat flux on surfaces. In the condensation heat transfer on wall surfaces, the size of the structure, material, and water–solid interaction are considered to play an important role. Widely analyzing the size of structures and their interaction with water is essential for understanding the micro- and nanostructure effects on water condensation. Therefore, in this study, we performed a molecular dynamics simulation to reveal the condensation mechanism of water droplets from vapor on nanoscale structures. In addition, we analyzed the contact angles in the static state in relation to the condensation types. The wall–water interaction parameter was changed in the range of hydrophilic to hydrophobic region.

## 2. Computational Methods 

We performed a molecular dynamics simulation to analyze the effect of the surface structure on the water wettability and condensation process. Coulombic and van der Waals interactions were treated as the intermolecular interactions. The TIP4P model [40], which is the four-site model, was employed as a water molecule model and the Lennard-Jones particle was used as the wall surface. To investigate the effect of adhesion force of wall on the wetting and condensation, the parameter *ε* was changed as shown in Table 1, *ε* = 19.74 kJ/mol (hydrophilic) to 0.06168 kJ/mol (hydrophobic). This approach that changes the *ε* parameter is similar to the previous molecular dynamics simulation in graphene [35]. The molecular size parameter was set to *σ* = 0.233 nm, the distance at which the intermolecular potential between the two particles is zero. The LJ parameter *ε* = 19.74 kJ/mol, the upper value of *ε*, and *σ* = 0.233 nm are same values used for copper, a typical hydrophilic metal [27]. The cutoff radius for the van der Waals interaction was 1.3 nm, and timestep was set to 2.0 fs. The Ewald method was employed for the calculation of Coulombic interaction. The calculation was performed using GROMACS [41,42]. The simulation was performed under constant number of molecules, volume, and temperature (NVT). The temperature was controlled by Nose–Hoover thermostat [43,44].

**Figure 2:**
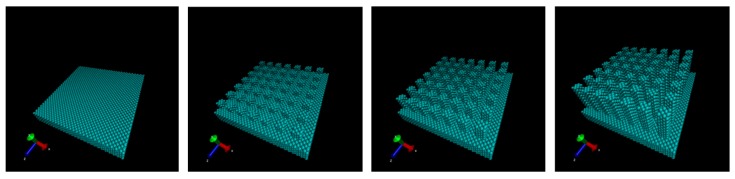
Prepared surface structure. (flat, asperity height 0.724 nm, asperity height 1.448 nm, and asperity height 2.896 nm).

Three patterns of a nanostructure with different heights and a flat surface were employed as the microfabricated surface as shown in Figure 2. The heights of the nanostructure were set as multiples of the length of the lattice constant of fcc copper 0.362 nm (0.724 nm, 1.448 nm, and 2.896 nm, respectively). The surface wall was a 10 nm square, 1.448 nm in thickness, under periodic boundary condition. The length of the height direction of the simulation box was 50 nm. 

**Figure 3:**
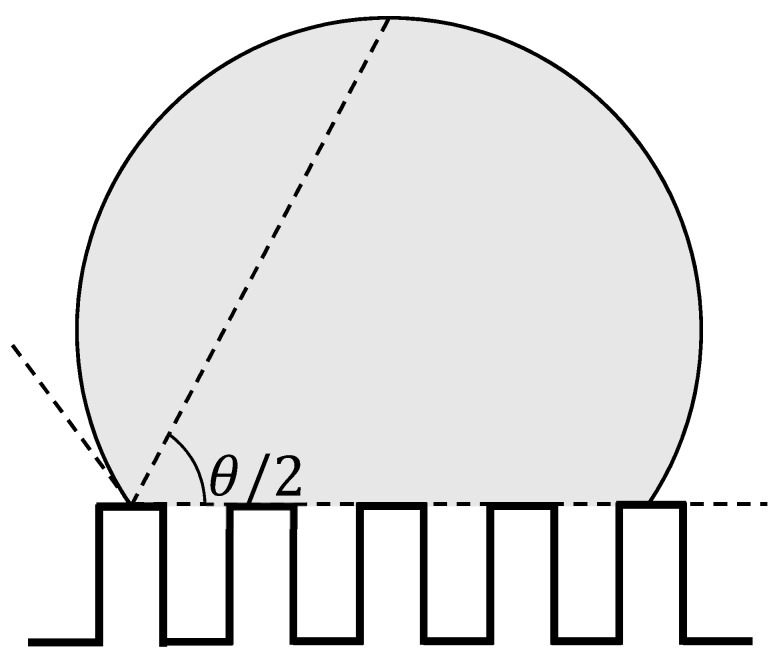
Schematic diagram of contact angle *θ* measurement on nanostructures by half angle method.

The contact angle of droplets deposited on the wall was measured using the half-angle method [45]. The schematic figure of the determination of contact angle *θ* on the nanosurface in this study is illustrated as Figure 3. The contact angle was determined as the angle of the line from the triple phase point to the apex of the drop and the line of the top of the wall. In the initial state of the simulation, a droplet was placed 0.5 nm from the wall and its natural adsorption on the wall was analyzed. To understand the effect of the size of the droplet on the wall, two diameters of the droplet, about 5 nm (2259 water molecules) and about 6 nm (3787 water molecules) were explored. The contact angle was calculated after 4 ns to reach the equilibrium at 300 K.

**Figure 4:**
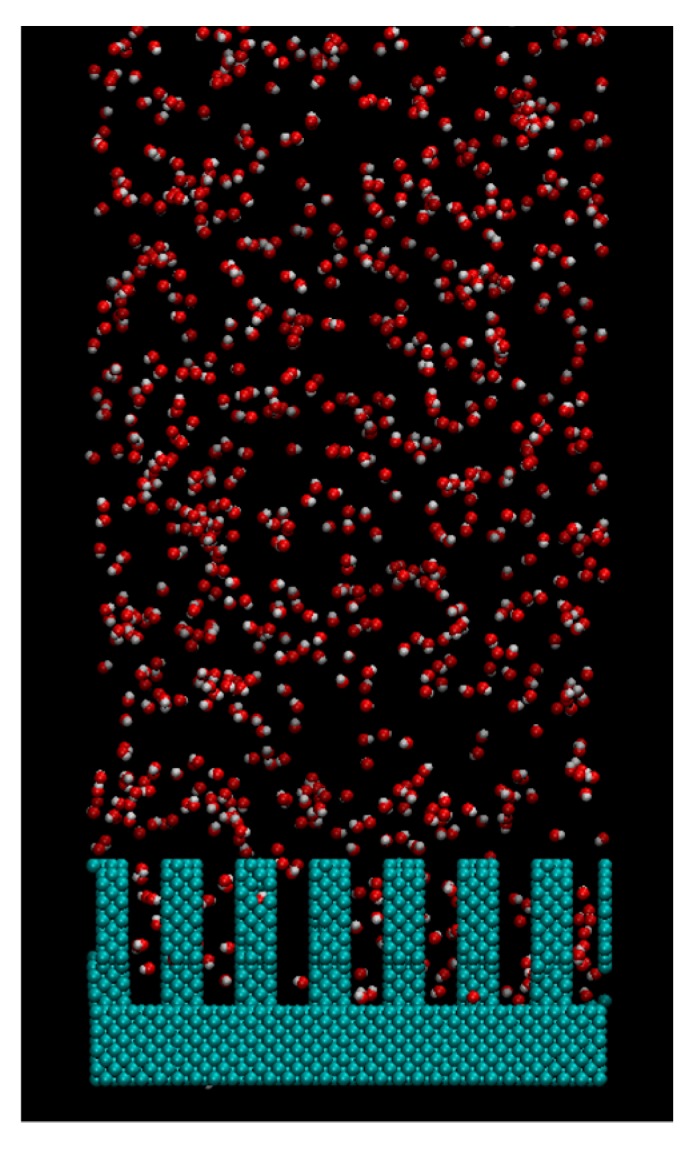
Snapshot of water vapor and nanostructure prepared as the initial condition of the condensation simulation.

The condensation process of water vapor on the nanostructured surface was calculated by 5 ns simulation under 300 K. The initial structure of the calculation was prepared by the 2 ns calculation under 600 K for 2259 water molecules on the surface as shown in Figure 4. We changed the LJ potential parameter *ε* in three patterns, 19.74 kJ/mol, 1.974 kJ/mol, 0.06168 kJ/mol. Three calculations were performed for each condition to obtain average values.

## 3. Results and Discussions

**Figure 5:**
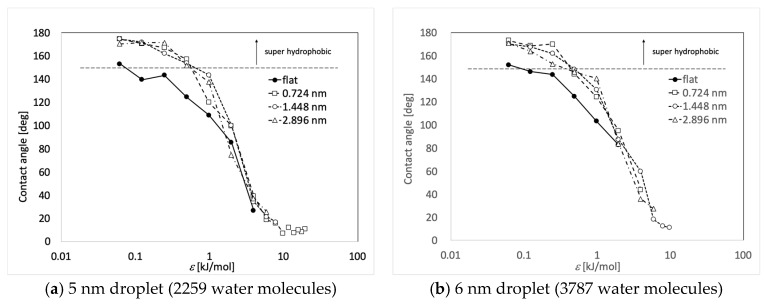
Relation between the water–wall interaction and the contact angle. The surface with asperity resulted in the increase of the contact angle.

**Figure 6:**
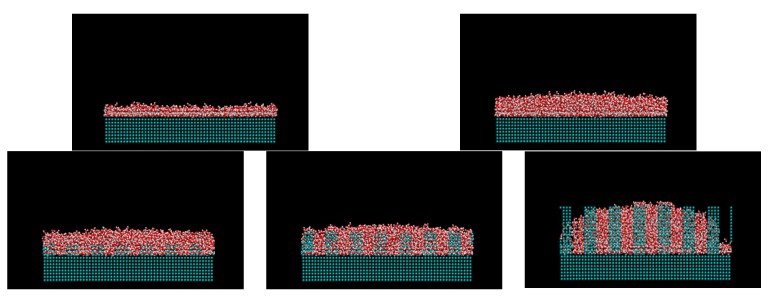
Snapshot of the water film on the flat and nanostructured surfaces with the Lennard-Jones parameter *ε* = 19.74 kJ/mol (the left upper: 2259 water molecules, others: 3787 water molecules).

Figure 5 and Table 2 and Table 3 show the calculated contact angles when the droplets with sizes of 5 nm and 6 nm were on the wall surface. Both contact angle dependence on the water–wall interaction and height of pillar showed similar trends with the previous study on graphene [35]. In Table 2 and Table 3, the numbers on the gray background correspond to the Wenzel state, the numbers without any background represent the Cassie-Baxter state, while no number means no droplet. The contact angle was determined as the angle at the top of the surface of asperity. The part where the angle could not be calculated corresponds to the area where the solid–liquid part was not formed stably because the liquid spread over the entire surface. Figure 6 shows a snapshot of the case, where water molecules spread into a film and the contact angle could not be determined on the plane of *ε* = 19.74 kJ/mol. When the adsorption force of the wall was large, water spread into a film on both the flat and uneven surfaces. Even when there was unevenness, water adhered to the available contact area and did not form droplets. The process of forming such a liquid film is consistent with previous molecular simulations on copper surfaces [39]. This behavior is similar to that of macroscopic films spread thinly when droplets are adsorbed on a hydrophilic surface [46,47]. Two layers of water molecules were found on the surface in the case of a film formed by 5 nm droplets on the flat surface. The hydrophilic surface adsorbed water molecules and aligned them. The boundary *ε* for the wetting state changing from Wenzel to Cassie-Baxter was 1.974 or 0.9869 kJ/mol, depending on the pillar height. There was no difference in the size of the droplets.

**Figure 7:**
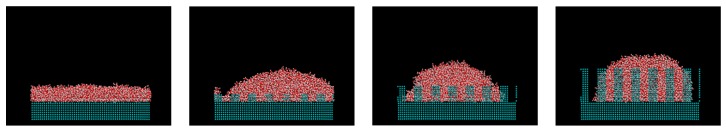
Droplets on nanostructures with *ε* = 3.948 kJ/mol.

**Figure 8:**
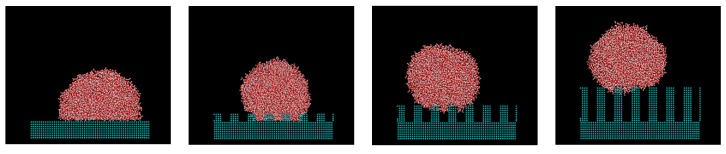
Droplets on nanostructures with *ε* = 0.9869 kJ/mol.

Figure 7 shows the change in the wetting state when asperity changed for a wall adsorption force of *ε* = 3.948 kJ/mol and a droplet with a diameter of 6 nm. While the liquid spreads over the whole plane on a flat wall, the Wenzel state is manifested by adding unevenness. Similarly, Figure 8 shows how the Wenzel and Cassie states are switched depending on the size of unevenness for the wall adsorption energy *ε* = 0.9869 kJ/mol. Overall, the contact angles were increased by the nanostructures by about 10° to 40°. The contact angle tended to increase with asperity and as the interaction between water molecules and the wall surface decreased. It was also possible to estimate how the liquid film, Wenzel state, and Cassie state changed depending on the surface adsorption force and nanoscale unevenness size when droplets adhered to the solid surface.

**Figure 9:**
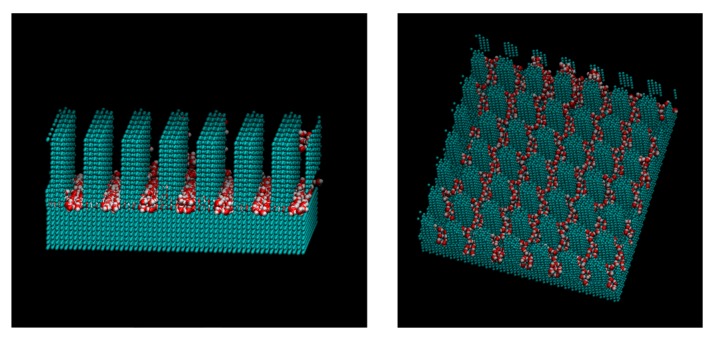
Snapshot of water molecules during the condensation on the *ε* = 19.74. kJ/mol surface from the different viewpoints. The water molecules are adsorbed into the pillar and formed water film.

**Figure 10:**
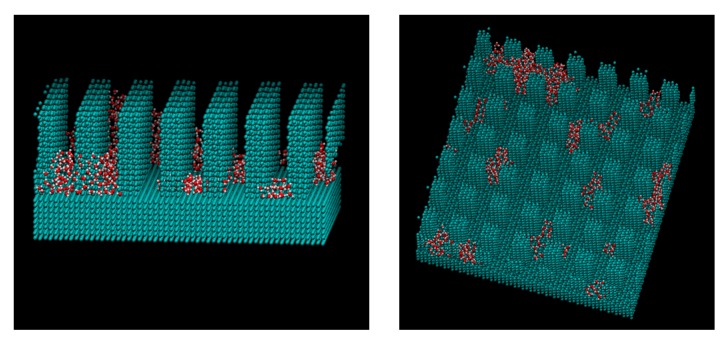
Snapshot of water molecules during the condensation on the *ε* = 1.974. kJ/mol surface from the different viewpoints. The water molecules formed small clusters in the pillar.

**Figure 11:**
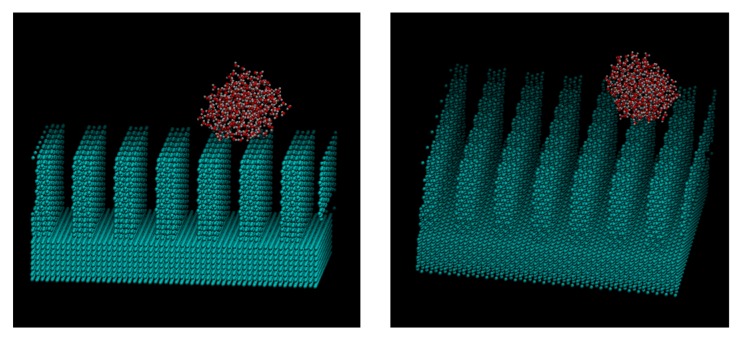
Snapshot of water molecules during the condensation on the *ε* = 0.06168. kJ/mol surface from the different viewpoints. The water droplet did not enter the nanostructure.

**Figure 12:**
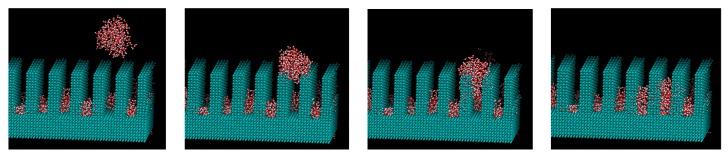
Absorption behavior of water droplets intruding into the inside of asperities with *ε* = 1.976 kJ/mol. (0 s, 35 ps, 90 ps, 350 ps).

**Figure 13:**
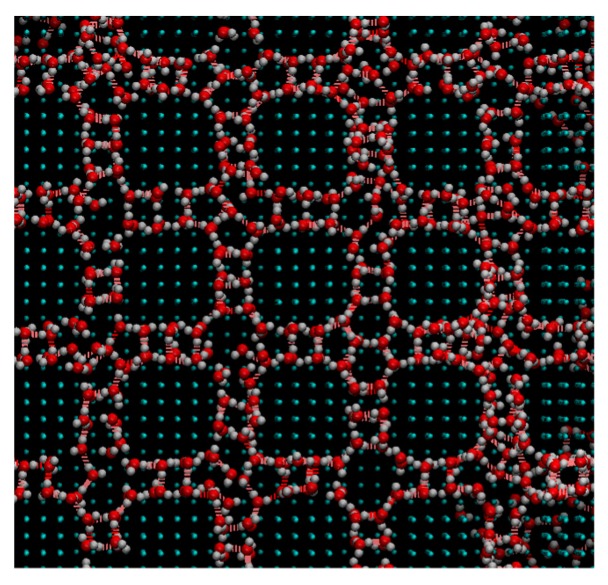
Snapshot of the two-dimensional structure of water observed in a nanostructured surface with *ε* = 19.74. kJ/mol.

Figure 9, Figure 10 and Figure 11 show the appearance of the convex surface in the case of *ε* = 19.74 kJ/mol, 1.974 kJ/mol, and 0.06168 kJ/mol. These parameters correspond to the liquid film, Wenzel state, and Cassie-Baxter state, respectively, in the calculation of the water droplets. When water molecules cooled down, both condensation near the surface and in the vapor could be observed. Figure 9 shows the snapshot of condensation with a strong water–surface interaction. Condensed water molecules formed a liquid film and uniformly attached to the inner wall of asperities. The surface of the hydrophilic nanostructure was wet in the initial stage of the condensation process. In addition, the small water droplets formed in the water vapor were observed to be absorbed into the asperity surface. Figure 12 shows the absorption behavior of water droplets intruding into the inside of asperities. It was found that the time scale of droplet adsorption is several tens to hundreds of ps. In such a hydrophilic nanostructure, water molecules spreading thinly inside asperities formed an orderly structure different from a bulk liquid. Figure 13 illustrates a snapshot of the two-dimensional structure of water observed in the nanostructured surface. This type of ordered structure is unique to confined systems such as inside nanotubes and graphene plates [48,49]. These results indicate that water molecules in nanostructured hydrophilic metal surfaces form unusual phase structures similar to other confined systems. Figure 10 demonstrates condensation of water on a wall with a low interaction level. When the interaction level became smaller, smaller droplets gradually attached to the solid surface but were not uniformly spread. Several droplets formed and gradually integrated. Figure 11 demonstrates the case of condensation on a hydrophobic surface with a very small interaction level. Even when a small number of water molecules formed a few clusters within the asperity, they gradually discharged to the outside of the asperity. In the end, almost no water molecules were left inside the asperities, and the droplets were attached to the surface.

**Figure 14:**
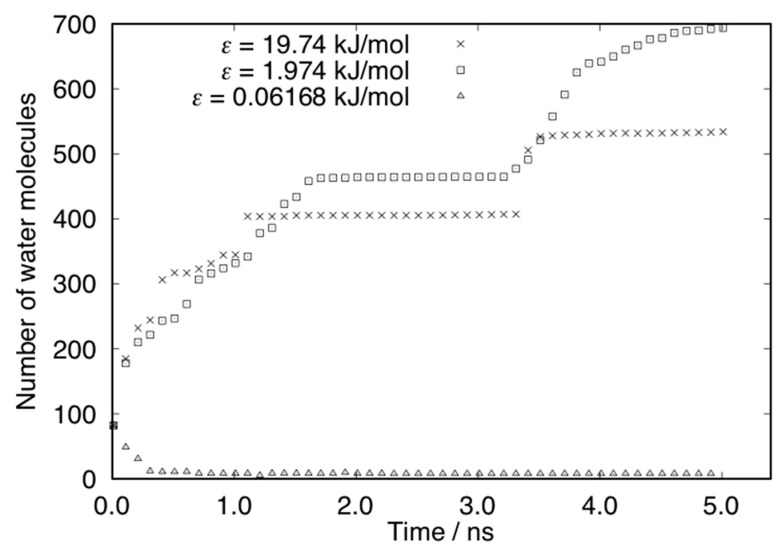
Number of water molecules in the nanostructure on surfaces.

**Figure 15:**
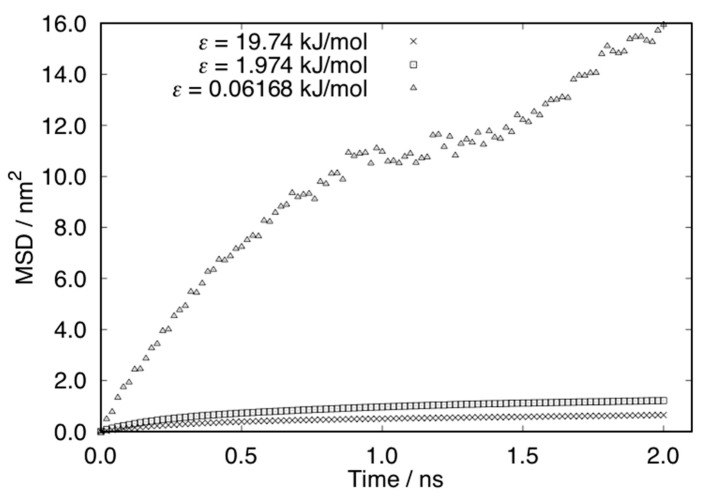
Mean square displacement (MSD) of water molecules in the nanostructure on surfaces.

Figure 14 shows the average number of water molecules inside the surface nanostructure for each case. The number of water molecules was increased for the cases of *ε* = 19.74 kJ/mol and *ε* = 1.974 kJ/mol by adsorption on the surface. For the first 1 ns, isolated water molecules near the surface adsorbed on the nanostructures continuously. After 1 ns, the increase in the number of water molecules showed jumps due to the adsorption of water droplet formed in the vapor phase. On the other hand, the number of water molecules was decreased in the case of *ε* = 0.06168 kJ/mol. After 1 ns, a small number of water molecules was trapped in the nanostructure. Figure 15 shows the mean square displacement (MSD) of the water molecules in the nanostructure. The MSD of the case of *ε* = 19.74 kJ/mol and *ε* = 1.974 kJ/mol is small because the water molecules on the surface were almost fixed or restricted in the droplet. The MSD for the case of *ε* = 0.06168 kJ/mol is much larger than the others. The small number of water molecules trapped in the nanostructure moved quickly on the surface.

As demonstrated here, the three types of condensation behavior—film, droplet, and discharge—appeared according to the difference in the strength of the surface interaction. In particular, when the adsorptive force was large, as is the case with copper, water molecules aggregated on the surface of the asperity and prepared a water film. The differences in the condensation behavior affected the condensation speed and diffusion of water molecules on the surface.

## 4. Conclusions

The impact of the wall nanostructure and adsorption force on the contact angle of droplets and condensation was analyzed using molecular dynamics simulation. As a result, the dependence of the contact angle and condensation behavior on the microfabrication shape and size of the wall was revealed. As the condensation behavior, the liquid film formation, droplet adsorption in the structure, and droplet discharge process were observed. The water molecules adsorbed on the surfaces showed little diffusion in the case of *ε* = 19.74 kJ/mol and *ε* = 1.974 kJ/mol. In addition, a two-dimensional structure of water molecules spread into the fine structure was observed.

## Figures and Tables

**Table 1:** The parameters of *ε* in the calculation. The *ε* = 19.74 kJ/mol is the case of copper.

Ratio	*ε* (kJ/mol)
1.0	19.74
0.9	17.76
0.8	15.79
0.7	13.82
0.6	11.84
0.5	9.869
0.4	7.895
0.3	5.921
0.2	3.948
0.1	1.974
1/20	0.9869
1/40	0.4935
1/80	0.2467
1/160	0.1234
1/320	0.06168

**Table 2:** Contact angle and wetting state of 5-nm droplets on each surface. The numbers on the gray background correspond to the Wenzel state, whereas the numbers without background correspond to the Cassie state.

*ε* (kJ/mol)	Flat	0.724 nm	1.448 nm	2.896 nm
19.74	-	10.6	-	-
17.76	-	8.1	-	-
15.79	-	10.0	-	-
13.82	-	7.5	-	-
11.84	-	11.8	-	-
9.869	-	7.0	-	-
7.895	-	15.8	16.7	-
5.921	-	18.5	21.1	25.3
3.948	26.5	39.0	34.3	37.2
1.974	85.4	99.7	99.9	74.4
0.9869	108.8	120.0	143.4	137.5
0.4935	124.4	157.1	153.7	151.8
0.2467	143.3	167.0	162.0	171.3
0.1234	139.6	170.5	171.9	171.4
0.06168	153.0	174.5	174.6	170.5

**Table 3:** Contact angle and wetting state of 6-nm droplets on each surface. The numbers on the gray background correspond to the Wenzel state, whereas the numbers without background correspond to the Cassie state.

*ε* (kJ/mol)	Flat	0.724 nm	1.448 nm	2.896 nm
19.74	-	-	-	-
17.76	-	-	-	-
15.79	-	-	-	-
13.82	-	-	-	-
11.84	-	-	-	-
9.869	-	-	10.9	-
7.895	-	-	12.3	-
5.921	-	-	18.1	27.4
3.948	-	44.0	59.6	36.0
1.974	82.6	95.0	87.3	83.8
0.9869	103.2	124.1	130.6	140.3
0.4935	124.6	144.1	148.2	145.9
0.2467	143.7	170.1	161.8	153.0
0.1234	146.3	168.5	167.5	163.7
0.06168	152.1	173.2	170.3	170.7
